# Targeting DGKα/PA axis inhibits tumor immune evasion and augments sensitivity to immunotherapy in gastrointestinal cancers

**DOI:** 10.1002/imt2.70120

**Published:** 2026-03-12

**Authors:** Jie Chen, Siqi Liu, Ting Peng, Fenglong Wang, Yuheng Zhu, Jingyuan Pang, Qingnan Wu, Yan Wang, Qimin Zhan

**Affiliations:** ^1^ Key Laboratory of Carcinogenesis and Translational Research (Ministry of Education/Beijing), Laboratory of Molecular Oncology Peking University Cancer Hospital & Institute Beijing China; ^2^ Peking University International Cancer Institute Peking University Beijing China; ^3^ Soochow University Cancer Institute Suzhou China; ^4^ Institute of Cancer Research Shenzhen Bay Laboratory Shenzhen China

## Abstract

Immune checkpoint inhibitors (ICIs) have shown promising antitumor efficacy in certain types of solid tumors. However, the efficacy of ICIs remains unsatisfactory owing to the dysregulation of signaling pathways in local tumor tissues. Here, we reveal that diacylglycerol kinase α (DGKα)‐derived phosphatidic acid (PA) directly binds to nuclear factor‐κB (NF‐κB) and enhances the transcriptional activity of NF‐κB to increase the expression of programmed cell death 1‐ligand 1 (PD‐L1) and facilitate the immune evasion of tumor cells and orchestrate immune microenvironment. Inhibition of DGKα activity decreases the intratumoral PD‐L1 level and induces cytotoxic T lymphocytes (CTLs) infiltration and resultantly enhances the antitumor efficacy of ICIs. Plasma PA can function as a biomarker to evaluate the efficacy of ICIs in gastrointestinal cancers. Overall, our results identify the DGKα/PA axis as a metabolic driver of immune evasion and CTLs exclusion, representing a promising target to enhance ICIs' efficacy in gastrointestinal cancer treatments.

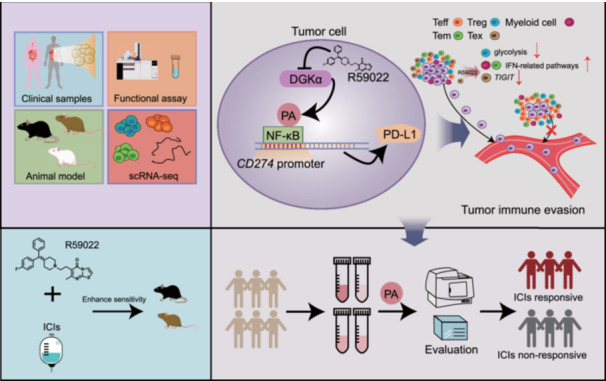

To the editor,

Despite significant progress in drug development and optimization of first‐line chemotherapy, the overall rate of patients with gastrointestinal cancer remains low [1, 2, 3, 4]. The molecular mechanisms underlying immune checkpoint inhibitor (ICI) non‐responsiveness remain unclear. Therefore, novel techniques combined with innovative treatment strategies and diagnostic biomarkers are urgently needed to bridge the gap between preclinical target discovery and the clinical efficacy of immunotherapy.

Diacylglycerol kinases (DGKs), a family of lipid kinases, catalyze the conversion of diacylglycerol (DAG) to phosphatidic acid (PA), which mediates cell growth and motility [5, 6, 7]. Notably, DGKα activates several signaling pathways to induce tumor malignancy [8, 9]. Specifically, the DGKα/PA axis can induce esophageal squamous cell carcinoma (ESCC) malignancy and cisplatin resistance by activating the nuclear factor‐κB (NF‐κB) pathway, effects that are reversed by the DGKα functional inhibitor‐R59022 [9, 10, 11, 12, 13]. However, the exact mechanism by which PA activates signaling proteins and induces tumor progression and immune evasion requires further investigation.

The molecular mechanisms underlying the interaction between lipids and the biological activities of cluster of differentiation 8^+^ (CD8^+^) T cells are underexplored. Inhibition of tumor cells‐produced sphingolipids effectively facilitates the anti‐growth effects of natural killer (NK) and CD8^+^ T cells [14]. Microbiota produces the short‐chain fatty acid butyrate to facilitate cellular metabolism and enhance the antitumor function of activated CD8^+^ T cells [15]. Here, we demonstrate that DGKα‐derived PA binds to NF‐κB p65 to induce programmed cell death 1‐ligand 1 (PD‐L1) expression. DGKα inhibition orchestrates immune microenvironment and enhances CD8^+^ T cell infiltration into tumor tissues to inhibit tumor malignancy. Specifically, plasma PA serves as a biomarker for ICIs response in gastrointestinal tumors.

## THE EXPRESSION OF DGKα POSITIVELY CORRELATES WITH THAT OF PD‐L1 IN GASTROINTESTINAL CANCERS

We examined the correlation between DGKα and PD‐L1 expression in several types of gastrointestinal tumors using sequential slices immunohistochemistry (IHC) assay (Figure S1A). As results shown in Figure S1B,C and Table S1, the expression of DGKα was positively correlated with that of PD‐L1 in gastrointestinal tumors, including ESCC, adenocarcinoma of the gastroesophageal junction, gastric cancer, colon cancer, rectal cancer, hepatocellular carcinoma, pancreatic cancer, and gallbladder cancer (Pearson *r* = 0.9091 in eight cancers, and *p* < 0.0001 in eight cancers).

## INTRATUMORAL DGKα/PA AXIS IS CRITICAL FOR PD‐L1 EXPRESSION

To evaluate whether DGKα regulates PD‐L1, we treated the indicated tumor cells with R59022 (25 or 50 μM) for 24 h. The results from quantitative enzyme‐linked immunosorbent assay (ELISA; Figure S2A) and immunoblotting assay (Figure S2B) showed that R59022 inhibited the expression of PD‐L1 in a dose‐dependent manner. We then depleted intratumoral *DGKA* using short hairpin ribonucleic acid (shRNA; Figure S2C) and found that *DGKA* depletion effectively suppressed the expression of PD‐L1 in tumor cells (ELISA, Figure S2D; immunoblotting assay, Figure S2E). PA (50 μM) recovered the expression of PD‐L1 in tumor cells harboring *DGKA* shRNA (Figure S2D,E) [8, 9, 10].

The human peripheral blood mononuclear cell‐mediated inhibition of tumor growth assay was conducted to assess the effect of blocking the DGKα/PA axis on the T cell‐mediated antitumor efficacy. After co‐culturing with T cells, R59022 (50 μM) facilitated T cell‐mediated growth inhibitory effect on indicated tumor cells (Figure S2F).

## DGKα/PA AXIS STIMULATES THE PD‐L1 EXPRESSION BY DIRECTLY INTERACTING WITH NF‐κB P65 AND ACTIVATING ITS TRANSCRIPTIONAL ACTIVITY

**FIGURE 1:**
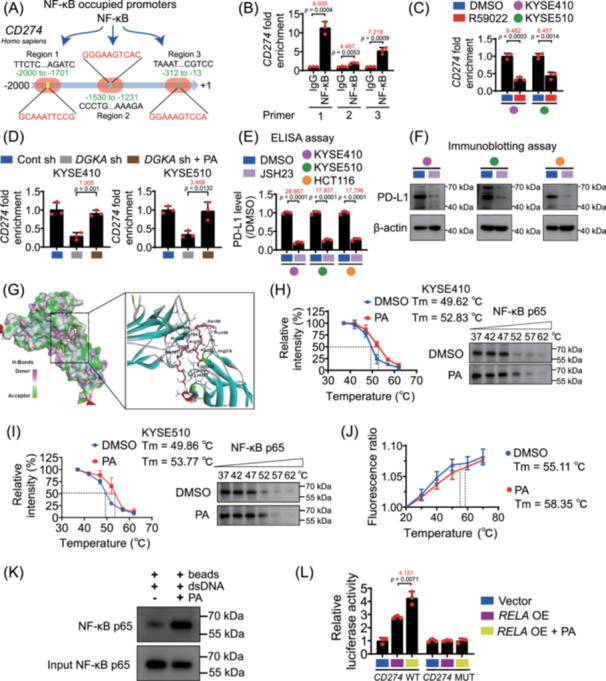
DGKα/PA axis stimulates NF‐κB p65‐mediated *CD274* transcriptional activation. (A) The diagram shows that NF‐κB p65 occupies the GCAAATTCCG (region 1), GGGAAGTCAC (region 2) and GGAAAGTCCA (region 3) regions of *CD274* promoter. (B) Chromatin immunoprecipitation (ChIP) assay results show the binding of NF‐κB p65 and *CD274* promoter regions in KYSE410 cells. (C) ChIP assay results show that R59022 (50 µM) blocks the NF‐κB p65‐interacted *CD274* promoter region in KYSE410 and KYSE510 cells. (D) ChIP assay results show that knocking down DGKα by shRNA inhibits the NF‐κB p65‐interacted *CD274* promoter region and adding PA (50 µM) recovers the interaction between NF‐κB p65 and *CD274* promoter region in KYSE410 and KYSE510 cells. Results from quantitative ELISA (E) and immunoblotting (F) assays show that NF‐κB p65 inhibitor‐JSH23 (10 µM) suppresses the expression of PD‐L1 in KYSE410, KYSE510 and HCT116 cells. (G) The molecular docking result of PA and NF‐κB p65 (approximately Ala156‐Arg274). CETSA results show PA (50 µM) interacts with NF‐κB p65 in KYSE410 (H) and KYSE510 (I) cells from 37°C to 62°C. (J) The nanoDSF results show the Tm value of recombinant human NF‐κB p65 protein with or without PA (200 µM). (K) The biotin‐dsDNA pull‐down assay results indicate that PA (50 µM) enhances the binding of NF‐κB p65 and the promoter of *CD274*. (L) Luciferase reporter assay shows that PA (50 µM) facilitates NF‐κB p65‐induced *CD274* transcriptional activation. Data are represented as mean ± SD of *n* = 3 biologically independent samples. Statistical significance for (B, C, D, E, and L) was determined by an unpaired Student's *t*‐test. *p* values and size effect (Cohen's *d*, marked in red) are shown.

We explored that the mechanism of DGKα/PA axis controls NF‐κB‐induced PD‐L1 expression (Figure S3). We identified that NF‐κB bound to the promoter region of *CD274* (Figure 1A,B); this interaction was disrupted by R59022 (50 μM) and *DGKA* shRNA in tumor cells (Figure 1C,D). PA (50 μM) restored this interaction in the DGKα‐depleted tumor cells (Figure 1D). Furthermore, the NF‐κB inhibitor JSH23 (10 μM) inhibited the expression of PD‐L1 (Figure 1E,F), and PA failed to induce PD‐L1 in *RELA proto‐oncogene* (*RELA*)‐silenced tumor cells, demonstrating the existence of PA/NF‐κB p65/PD‐L1 axis in tumor cells (Figure S4A,B).

PA directly bound to the amino acid sites (approximately Ala156 to Arg274, especially H181, P182, F184, P189, Q220, K221, or R274) of NF‐κB p65 protein (PDB ID: 1NFI) (Figure 1G). The results of the cell thermal shift assay (CETSA) and nano‐differential scanning fluorimetry assay (nanoDSF) showed that PA directly interacted with NF‐κB p65 at the cellular or in vitro level (Figure 1H–J). CETSA results showed that the interaction between PA and NF‐κB p65 was effectively disrupted by specific mutants (H181A, P182A, F184A, P189A, Q220A, K221A, or R274A) in KYSE410 cells (Figure S5A). Biolayer interferometry (BLI) assay confirmed that the mutated peptides significantly reduced the binding affinity of PA/NF‐κB p65 compared to the wild‐type peptides (Figure S5B). Biotin‐double strand deoxyribonucleic acid (dsDNA) pull‐down and luciferase reporter assays revealed that PA promoted NF‐κB p65 binding to region 1 of the *CD274* promoter (Figure 1K,L). Unlike complex cytokine cascades, we reveal that PA acts as a direct activator, binding NF‐κB p65 to induce PD‐L1 expression and provide novel mechanism of NF‐κB/PD‐L1 axis‐mediated tumor immune evasion.

## DGKα INHIBITION BLOCKS ESCC MALIGNANCY AND PROMOTES CYTOTOXIC T CELLS (CTL) INFILTRATION IN A HUMANIZED XENOGRAFT MOUSE MODEL OF ESCC

**FIGURE 2:**
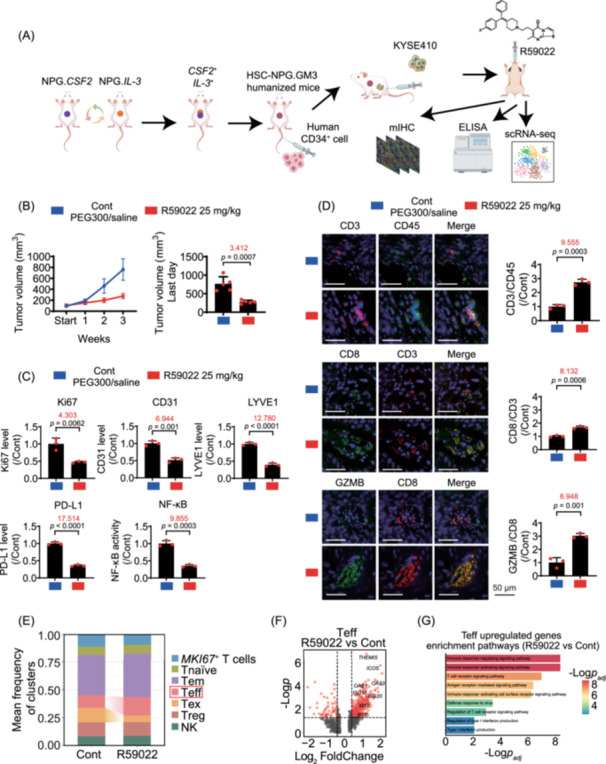
DGKα inhibitor R59022 suppresses tumor progression via inhibiting NF‐κB/PD‐L1 pathway and facilitates CTLs infiltration in humanized xenograft model. (A) The diagram shows the experimental process of establishing subcutaneous tumor‐bearing model and detection methods. (B) Changes of tumor volume within 3 weeks in R59022‐treated humanized mice harboring KYSE410 tumors (left panel). The tumor volume on the last day is indicated (right panel). (C) Results from quantitative ELISA assay show that R59022 inhibits the expression of Ki67, CD31, LYVE‐1, PD‐L1 and the activity of NF‐κB in KYSE410 tumors of humanized mice mode. (D) Results from multiplex staining assay show that R59022 increases the CD3^+^/CD45^+^, CD8^+^/CD3^+^ T cells and GZMB^+^CD8^+^ CTL cells infiltration in KYSE410 tumors of humanized mice model. cRNA‐seq results show that R59022 increases the Teff ratio in tumor tissues (E) and up‐regulates the amount of Teff‐related genes (F) and signaling pathways (G). Data are represented as mean ± SD of *n* = 5 (B) and 3 (C, D) biologically independent samples. Statistical significance for (B, C, and D) was determined by unpaired Student's *t*‐test. *p* values and size effect (Cohen's *d*, marked in red) are shown.

To study the functional interactions between human ESCC cells and T lymphocytes in vivo, we established a humanized xenograft model by transplanting human hematopoietic stem cells into NPG mice harboring human *colony‐stimulating factor 2* (*CSF2*) and *interleukin‐3* (*IL‐3*) genes (Figure 2A). Oral administration of R59022 (25 mg/kg/day) effectively inhibited the growth of KYSE410 tumors in humanized mice (Figure 2B). R59022 downregulated intratumoral proliferation marker protein Ki‐67 (Ki67), CD31, lymphatic vessel endothelial hyaluronan receptor‐1 (LYVE‐1), and PD‐L1 expressions and suppressed NF‐κB activity (Figure 2C). R59022 enhanced the infiltration of CD3^+^/CD45^+^ cells, CD8^+^/CD3^+^ T cells, and granzyme B^+^ (GZMB^+^) CD8^+^ CTLs in local KYSE410 tumors (Figure 2D). Interestingly, R59022 (25 mg/kg/day, orally) could inhibit the growth of KYSE410 tumor in BALB/c‐nu mice (Figure S6A,B). R59022 inhibits KYSE410 tumors in immune‐competent and ‐deficient mice, indicating that both tumor‐intrinsic and immune‐mediated antitumor mechanisms of DGKα inhibition.

Single‐cell RNA sequencing (scRNA‐seq) analysis of tumor tissues in this xenograft model revealed that R59022 inhibited many tumor‐promoting molecules in tumor cells (identified by *epithelial cell adhesion molecule* (*EPCAM*)), especially cell proliferation‐related molecules and some NF‐κB‐controlled genes, providing the panorama of DGKα‐controlled signaling networks in tumor cells (Figure S7A,B, and Table S2). Specifically, we hypothesize that PA promotes immune evasion not only by inducing PD‐L1 but also by remodeling the cytoskeleton. scRNA‐seq analysis identified seven NK/T cell subsets based on canonical markers (Figure S8A,B). R59022 increased the proportion of effector T cell (Teff) (Figure 2E). Results of differential expression and gene ontology (GO) analyses showed that *thymocyte selection‐associated* (*THEMIS*) and *inducible T‐cell co‐stimulator* (*ICOS*) were among the most significantly upregulated genes in R59022‐treated Teff, with upregulated genes showing an observable enrichment in pathways related to T cell activation and interferon (IFN) signaling (Figure 2F,G, and Table S3). A similar trend was observed in effector memory T cell (Tem), where R59022 appeared to enhance IFN‐related pathways (Table S4), while a reduction in the expression of the exhaustion marker *T‐cell immunoreceptor with Ig and ITIM domains* (*TIGIT*) was noted in exhausted T cell (Tex) (Figure S9). Furthermore, R59022 was associated with the downregulated expression of several glycolysis‐related genes, such as *enolase 1* (*ENO1*), *pyruvate kinase muscle* (*PKM*), and *phosphoglycerate kinase 1* (*PGK1*) in regulatory T cell (Treg) (Figure S10A,B and Table S5) [16]. In *monocyte differentiation antigen CD14* ^+^ (*CD14* ^+^) myeloid cells, we also observed a tendency toward the activation of IFN‐related pathways (Figure S11A,B and Table S6). Our results reveal that R59022 upregulated IFN‐related genes (ISGs) in T cells and myeloid cells, suggesting that it enhances IFN signaling to coordinate innate and adaptive immune response. The observed Teff activation and upregulation of ISGs in our study may possibly be associated with enhanced IFN signaling, while activation of IFN pathways in myeloid cells could further amplify Teff function, potentially forming a positive immunoregulatory circuit.

## DGKα/PA AXIS‐MEDIATED THE ACTIVATION OF NF‐κB/PD‐L1 PATHWAY IS INDEPENDENT OF PROTEIN KINASE C ζ (PKCζ)

Although PA has been reported to activate NF‐κB via PKCζ in melanoma cell line [17], we found that PKCζ could not evidently interact with NF‐κB p65 in KYSE410 and KYSE510 cells, even upon PA stimulation (Figure S12A), and the PKCζ inhibitor‐ζ‐Stat (1 μM) failed to block PA‐induced NF‐κB binding to the *CD274* promoter or affect the PD‐L1 level (Figure S12B,C).

## PLASMA PA SERVES AS A BIOMARKER FOR RESPONSE TO ICIS IN GASTROINTESTINAL CANCERS

To evaluate the clinical potential of plasma PA as a biomarker for ICIs response/non‐response in gastrointestinal cancers, we applied two patient cohorts (Table S7 and Table S8). Cohort 1 consisted of plasma and paired tumors collected from 21 ESCC patients, 48 gastric cancer patients, and 12 colon cancer patients. Cohort 2 comprised independent plasma samples from 98 ESCC patients, 140 gastric cancer patients, and 79 colon cancer patients.

Results of quantitative ELISA assay showed that ICIs responsive groups had significantly lower levels of plasma PA (Cohort 1 and 2) and intratumoral DGKα/PA (Cohort 1) compared to non‐responsive groups in three types of cancers (Figure S13A–C, Figure S14, and Table S7 and Table S8) [10, 18].

In non‐responsive groups (Cohort 1), we observed significant positive correlations among plasma PA and intratumoral DGKα/PA levels in three types of cancers (Figure S13D). The Pearson correlation coefficient and statistical *p‐*value of plasma PA and tumor tissue DGKα/PA in responsive groups from ESCC and gastric cancer (Cohort 1) were shown in Figure S15A–C. Specifically, in cohort 2, receiver operating characteristic curve (ROC) analysis indicated that plasma PA exhibited favorable diagnostic performance in three types of cancers (Figure S16A–C). Here, we first demonstrate that plasma PA serves as a biomarker for evaluating ICIs response in gastrointestinal cancers, and propose that with optimized detection strategies, PA could serve as a reliable biomarker for assessing tumor progression and therapeutic response.

## INTRATUMORAL DGKα/NF‐κB AXIS WAS POSITIVELY CORRELATED WITH NON‐RESPONSE TO ICIS IN GASTROINTESTINAL CANCERS

As shown in Figures S13A, S17A,B and Table S7, the levels of DGKα or NF‐κB p65 protein in the ICIs non‐responsive groups were higher than those in the ICIs‐responsive groups of three types of cancers (Cohort 1), evaluated by IHC assay. The expression of DGKα was positively correlated with that of NF‐κB p65 in the ICIs non‐responsive groups in three types of cancers (Cohort 1, Figure S17C).

## INHIBITION OF THE ACTIVITY OF THE DGKα/PA AXIS AMPLIFIES THE EFFICACY OF ANTI‐CYTOTOXIC T‐LYMPHOCYTE ASSOCIATED PROTEIN‐4 (CTLA‐4) ANTIBODY IN AN IMMUNE‐COMPETENT MOUSE MODEL

In the MC38 and MFC mouse gastrointestinal tumor model, R59022 (25 mg/kg/day, orally) increased CD8^+^ cells and decreased PD‐L1 expression in tumor tissues (Figure S18A,B). Combination of anti‐programmed cell death protein‐1 (PD‐1) antibody with anti‐CTLA‐4 antibody is an efficient strategy in immunotherapy [19, 20]. Based on the decreased PD‐L1 levels after R59022 treatment, we evaluated whether R59022 enhances the antitumor efficacy of mouse anti‐CTLA‐4 antibody (Figure S19A). As shown in Figure S19B and Figure S20A,B, R59022 increased the anti‐growth effect of the anti‐CTLA‐4 antibody (50 μg/mouse up to four times, i.p.). Results of quantitative ELISA and IHC assay in Figure S19C,D and Figure S21 showed that R59022 enhanced the inhibitory effect of the anti‐CTLA‐4 antibody on the expression of indicated tumor‐promoting markers.

Low dose of R59022 (12.5 mg/kg/day, orally) and anti‐CTLA‐4 antibody produced synergistically inhibitory effect on the growth of indicated tumors (The Q values were 1.27 and 1.39 in MC38 and MFC, Figure S22A–D). Specifically, IHC results showed that R59022 (12.5 mg/kg/day) effectively enhanced the inhibitory effect of anti‐CTLA‐4 antibody on Treg level and the expression of CTLA‐4 and Ki67 (Figure S22E). Hematoxylin and eosin staining (H&E) of the major organs from MC38 or MFC tumor‐bearing mice revealed no obvious morphological changes across the indicated groups (Figure S23A,B). Collectively, R59022 synergistically enhances anti‐CTLA‐4 antibody efficacy by orchestrating immune microenvironment and promoting T cell‐mediated antitumor response, providing novel strategy to develop the combination of antitumor agents.

In conclusion, DGKα drives immune evasion via through NF‐κB/PD‐L1 signaling. Its inhibition orchestrates immune microenvironment to enhance ICIs efficacy, while Plasma PA evaluates ICIs response in gastrointestinal cancers.

## AUTHOR CONTRIBUTIONS

**Jie Chen**: Methodology; data curation; supervision; validation; investigation; writing—original draft; writing—review and editing; conceptualization; funding acquisition. **Siqi Liu**: Methodology; validation; investigation; visualization; writing—original draft. **Ting Peng**: Data curation; formal analysis; visualization. **Fenglong Wang**: Methodology; investigation; validation. **Yuheng Zhu**: Investigation; methodology; validation. **Jingyuan Pang**: Investigation; methodology; validation. **Qingnan Wu**: Investigation; methodology. **Yan Wang**: Investigation; methodology. **Qimin Zhan**: Supervision; writing—review and editing; funding acquisition; resources; project administration. All authors have read the final manuscript and approved it for publication.

## CONFLICT OF INTEREST STATEMENT

The authors declare no conflicts of interest.

## ETHICS STATEMENT

All animal procedures were approved by the Institutional Review Board of Peking University Cancer Hospital & Institute (EAEC 2024‐25). All clinical samples preparation procedures were approved by the Institutional Review Board of Peking University Cancer Hospital & Institute (2024KT176).

## Supporting information


**Figure S1:** The clinical correlation between DGKα and PD‐L1 in gastrointestinal cancers.
**Figure S2:** DGKα Inhibition blocks PD‐L1 expression and enhances the tumor‐inhibitory effect of T cells.
**Figure S3:** Diagram showing the approach and protocol to validate PA‐induced NF‐κB p65 transcriptional activity.
**Figure S4:** NF‐κB is critical for PA‐mediated PD‐L1 expression.
**Figure S5:** PA interacts with the specific sites of NF‐κB p65.
**Figure S6:** The growth inhibitory effect of R59022 on the KYSE410 tumor in BALB/c‐nu mice.
**Figure S7:** Differential expression of tumor‐promoting genes between control and R59022‐treated KYSE410 tumor.
**Figure S8:** Seven NK/T cell subsets clustering and markers expression in NK/T cell subsets.
**Figure S9:** R59022 inhibits the expression of Tex biomarker‐*TIGIT*.
**Figure S10:** Downregulation of glucose metabolism‐related pathways and genes in R59022‐treated Treg.
**Figure S11:** Upregulation of IFN‐related pathways and genes in R59022‐treated myeloid cells.
**Figure S12:** PKCζ is not involved in the DGKα/PA axis‐mediated PD‐L1 expression.
**Figure S13:** The expression of PA in plasma and paired tumor tissues and the correlation between plasma PA and tissue DGKα/PA axis in ICIs‐treated gastrointestinal cancers.
**Figure S14:** The expression of DGKα in tumor tissues in ICIs‐treated responsive and non‐responsive gastrointestinal cancers.
**Figure S15:** Correlation between plasma PA, tissue PA and tissue DGKα in ICIs‐treated responsive ESCC and GC patients.
**Figure S16:** ROC curve shows the effect of plasma PA on evaluating immunotherapy response.
**Figure S17:** The expression of DGKα and NF‐κB in ICIs‐treated responsive and non‐responsive gastrointestinal tumor tissues and the correlation between DGKα and NF‐κB in non‐responsive gastrointestinal tumor tissues.
**Figure S18:** CD8 and PD‐L1 levels in MC38 or MFC tumor tissues from R59022‐treated MC38 and MFC subcutaneous tumor‐bearing mice model.
**Figure S19:** Combined administration of R59022 and anti‐CTLA‐4 antibody in MC38 and MFC subcutaneous tumor‐bearing mice model.
**Figure S20:** The change of tumor volume in MC38 and MFC subcutaneous tumor‐bearing mice model.
**Figure S21:** Tumor markers expression in MFC subcutaneous tumor‐bearing mice model tumor tissues.
**Figure S22:** Combined administration of low dose R59022 and anti‐CTLA‐4 antibody in MC38 and MFC subcutaneous tumor‐bearing mice model.
**Figure S23:** The combination of R59022 and anti‐CTLA‐4 antibody does not induce obvious systemic toxicity in xenograft mouse model.

**Table S1:** The patient information of eight gastrointestinal cancers.
**Table S2:** R59022‐treated tumor cell downregulated genes.
**Table S3:** R59022‐treated Teff cell upregulated genes enrichment pathways.
**Table S4:** R59022‐treated Tem upregulated genes enrichment pathways.
**Table S5:** R59022‐treated Treg downregulated genes enrichment pathways.
**Table S6:** R59022‐treated myeloid upregulated genes enrichment pathways.
**Table S7:** ICIs responsive and non‐responsive patients' information of ESCC, CC and GC.
**Table S8:** Multiple linear regression analysis of Table S7.

## Data Availability

All the sequencing data have been uploaded to the National Genomics Data Center Biological Project Library with ID: PRJCA040528 (https://ngdc.cncb.ac.cn/bioproject/browse/PRJCA040528). The data and scripts used are saved in GitHub (https://github.com/Ting-PKU/DGKA_inhibition_project). Supplementary materials (methods, figures, tables, graphical abstract, slides, videos, Chinese translated version, and update materials) may be found in the online DOI or iMeta Science http://www.imeta.science/.
