# Supplementary material for: Targeting DGKα/PA axis inhibits tumor immune evasion and augments sensitivity to immunotherapy in gastrointestinal cancers
Source: Imeta. 2026 Mar 12;5(2):e70120. doi: 10.1002/imt2.70120 (PMC13147932; doi:10.1002/imt2.70120)
Supplement: Supplementary file 1 — Figure S1: The clinical correlation between DGKα and PD‐L1 in gastrointestinal cancers. Figure S2: DGKα Inhibition blocks PD‐L1 expression and enhances the tumor‐inhibitory effect of T cells. Figure S3: Diagram showing the approach and protocol to validate PA‐induced NF‐κB p65 transcriptional activity. Figure S4: NF‐κB is critical for PA‐mediated PD‐L1 expression. Figure S5: PA interacts with the specific sites of NF‐κB p65. Figure S6: The growth inhibitory effect of R59022 on the KYSE410 tumor in BALB/c‐nu mice. Figure S7: Differential expression of tumor‐promoting genes between control and R59022‐treated KYSE410 tumor. Figure S8: Seven NK/T cell subsets clustering and markers expression in NK/T cell subsets. Figure S9: R59022 inhibits the expression of Tex biomarker‐TIGIT. Figure S10: Downregulation of glucose metabolism‐related pathways and genes in R59022‐treated Treg. Figure S11: Upregulation of IFN‐related pathways and genes in R59022‐treated myeloid cells. Figure S12: PKCζ is not involved in the DGKα/PA axis‐mediated PD‐L1 expression. Figure S13: The expression of PA in plasma and paired tumor tissues and the correlation between plasma PA and tissue DGKα/PA axis in ICIs‐treated gastrointestinal cancers. Figure S14: The expression of DGKα in tumor tissues in ICIs‐treated responsive and non‐responsive gastrointestinal cancers. Figure S15: Correlation between plasma PA, tissue PA and tissue DGKα in ICIs‐treated responsive ESCC and GC patients. Figure S16: ROC curve shows the effect of plasma PA on evaluating immunotherapy response. Figure S17: The expression of DGKα and NF‐κB in ICIs‐treated responsive and non‐responsive gastrointestinal tumor tissues and the correlation between DGKα and NF‐κB in non‐responsive gastrointestinal tumor tissues. Figure S18: CD8 and PD‐L1 levels in MC38 or MFC tumor tissues from R59022‐treated MC38 and MFC subcutaneous tumor‐bearing mice model. Figure S19: Combined administration of R59022 and anti‐CTLA‐4 antibody in MC38 and MFC su [file IMT2-5-e70120-s002.docx]

**Supporting information to**

**Targeting DGKα/PA axis inhibits tumor immune evasion and augments sensitivity to immunotherapy in** **gastrointestinal cancers**

**Running title:** Targeting DGKα/PA axis sensitizes gastrointestinal cancers to immunotherapy

Jie Chen^1,2,3#*^, Siqi Liu^1,2#^, Ting Peng^1,2#^, Fenglong Wang^1,2^, Yuheng Zhu^1,2^, Jingyuan Pang^1^, Qingnan Wu^1,2^, Yan Wang^1,2^, Qimin Zhan^1,2,3,4*^

^1^Key Laboratory of Carcinogenesis and Translational Research (Ministry of Education/Beijing), Laboratory of Molecular Oncology, Peking University Cancer Hospital & Institute, Beijing 100142, China

^2^Peking University International Cancer Institute, Peking University, Beijing 100191, China

^3^Soochow University Cancer Institute, Suzhou 215000, China

^4^Institute of Cancer Research, Shenzhen Bay Laboratory, Shenzhen 518107, China

^*^Correspondence: [cj_blue@126.com](mailto:cj_blue@126.com) (Jie Chen); [zhanqimin@bjmu.edu.cn](mailto:zhanqimin@bjmu.edu.cn) (Qimin Zhan)

^#^ These authors contribute equally to this work

**Methods and materials**

**Antibodies**

DGKα (Cat # 11547-1-AP, RRID: AB_2245857), NF-κB p65 (Cat # 10745-1-AP, RRID: AB_2178878) and PD-L1 (Cat # 17952-1-AP, RRID: AB_10597552) antibodies were purchased from Proteintech. PKCζ antibody (Cat # A23777) was obtained from ABclonal. CD3 antibody (Cat # ZM-0417, RRID: AB_2890105, Zsbio), CD45 antibody (Cat # ZM-0813, Zsbio), CD8 antibody (Cat # ZA-0508, Zsbio), GZMB antibody (Cat # TA505931, RRID: AB_2623479, ThermoFisher) were used for multiplex staining assay. *MYC* proto-oncogene, bHLH transcription factor (c-Myc) antibody (Cat # 10828-1-AP, RRID: AB_2148585, Proteintech), Ki67 antibody (Cat # A20018, RRID: AB_3065688, ABclonal), high mobility group box 1 (HMGB1) antibody (Cat # 10829-1-AP, RRID: AB_2232989, Proteintech), matrix metalloproteinase-1 (MMP1) antibody (Cat # 10371-2-AP, RRID: AB_2297741, Proteintech), MMP2 antibody (Cat # A6247, RRID: AB_2766854, ABclonal), MMP7 antibody (Cat # 10374-2-AP, RRID: AB_2144452, Proteintech), forkhead box P3 (Foxp3) (Cat # 85128-6-RR, RRID: AB_3718551, Proteintech), vascular endothelial growth factor A (VEGFA) antibody (Cat # A0280, RRID: AB_2757092, ABclonal), VEGFC antibody (Cat # A12530, RRID: AB_2759371, ABclonal) and CTLA-4 antibody (Cat # 30648-1-AP, RRID: AB_3086380, Proteintech) were used for IHC assay in xenograft mice harboring MC38 and MFC tumors.

**Cell culture**

KYSE410 (JCRB1419, from Dr. Y. Shimada, JCRB Cell Bank), KYSE510 (JCRB1436, from Dr. Y. Shimada, JCRB Cell Bank) and HCT116 (Cat # CCL-247, RRID: CVCL_0291, from ATCC) cells were used to evaluate the expression of PD-L1 in the case where DGKα was inhibited and knocked down. Specifically, R59022 and shRNAs were employed to inhibit and knock down the activity and expression of DGKα in KYSE410, KYSE510 and HCT116 cells. The sequences of shRNAs were as follows [1]:

*DGKA* shRNA1: AGACACCTAAGCCTGGCACTGTTTCAATC;

*DGKA* shRNA2: GACAGGTGTTCAACCTCCTAAAGGATGGT.

The small interfering RNA (siRNA) sequences for *RELA* knockdown in indicated tumor cells were as follows:

*RELA* siRNA1: GATTGAGGAGAAACGTAAA;

*RELA* siRNA2: CCCACGAGCTTGTAGGAAA.

All cell lines used in present study were authenticated and have not been previously reported as misidentified or contaminated.

**ELISA assay evaluating PD-L1 expression**

ELISA assay was applied to detect level of PD-L1. The KYSE410 tumor tissues and KYSE410, KYSE510, and HCT116 cells were crushed and lysed on ice by RIPA lysis buffer with protease/phosphatase inhibitor cocktail for 30 min. Then, the lysates were centrifuged, and supernatants were collected for ELISA assay. The experiment was conducted in accordance with the instructions of the kit (Cat # ml952111V, mlbio).

**Immunoprecipitation and immunoblotting assay**

Cell lysates were prepared in NP40 buffer with protease/phosphatase inhibitors and centrifuged (12000 *g*, 20 min, 4 ℃). Supernatants were incubated with NF-κB p65 antibody and protein A/G beads overnight at 4 ℃. Beads were washed with cold lysis buffer, boiled in loading buffer for 5 min, and analyzed by immunoblotting.

For immunoblotting, samples were resolved by sodium dodecyl sulfate-polyacrylamide gel electrophoresis (SDS-PAGE) and transferred to polyvinylidene fluoride (PVDF) membranes, which were then blocked with nonfat milk in PBST (1 h), incubated with primary antibodies (overnight, 4 ℃), followed by secondary antibodies (1 h, room temperature). Signals were visualized using chemiluminescence.

**Chromatin immunoprecipitation (ChIP) assay**

The indicated cells were cross-linked with 1% formaldehyde (Cat # F8775, Sigma), lysed, and sonicated. ChIP was performed using NF-κB p65 antibody (Cat # 8242, RRID: AB_10859369, CST). Rabbit IgG was used as a negative control. NF-κB p65 antibody-bound DNA was quantified by real-time polymerase chain reaction (RT-PCR). The primer sequences (synthesized by Shanghai Shenggong Biotechnology Company) used were as follows:

*CD274* primer 1 (region 1), Forward: 5’- CGAATCCTCACATTACTAATACGCA -3’;

Reverse: 5’- AGTGTTCAGGGTCTACCTAAATGAT -3’.

*CD274* primer 2 (region 2), Forward: 5’- CTATATTTTCCTAGAGGTCACAGTC -3’;

Reverse: 5’- TGAGGTCACATAGTAGAATAGAGC -3’.

*CD274* primer 3 (region 3), Forward: 5’- GAAACTCTTCCCGGTGAAAATC -3’;

Reverse: 5’- CCAAGGCAGCAAATCCAG -3’.

**T cell-mediated inhibition of tumor growth assay**

T cell-mediated inhibition of tumor growth assay was exerted as previously described [2,3]. Briefly, primary human T cells isolated from healthy human peripheral blood were activated with a human CD3/CD28 T cell activator (Cat # 10970, STEMCELL Technologies) and IL-2 (Cat # AF-200-02, PeproTech; 10 ng/mL). KYSE410, KYSE510, or HCT116 cells were seeded at a density of 2,000 cells per well with or without R59022 (50 μM). Activated T cells were co-cultured with tumor cells at a ratio of 10:1. After 48 h, the cells were washed with phosphate-buffered saline solution (PBS) and analyzed using a cell counting kit-8 (CCK-8) according to the manufacturer’s instructions.

**Xenograft model**

All animal procedures were approved by Institutional Review Board of Peking University Cancer Hospital & Institute (EAEC 2024-25). Female (4-week) HSC-NPG.GM3 humanized mice were applied to evaluate the inhibitory effect of R59022 on tumor malignancy. Mouse embryos were respectively microinjected with transgenic vector harboring with human *CSF2* or *IL-3* genes and NPG. human granulocyte-macrophage colony-stimulating factor (hGM-CSF) and NPG. hIL-3 mice were hybridized to obtain transgenic homozygous mouse strain (NPG.GM3 humanized mice). KYSE410 cells (1 × 10^6^/mouse) were subcutaneously injected into the humanized mice, and when the tumor volume reached approximately 100 mm^3^, mice were orally treated with R59022 (25 mg/kg/day). The formula for evaluating tumor volume was based on our previous study [1]. After 3 weeks, the tumors were collected and subjected to scRNA-seq, multiplex staining, and quantitative ELISA assays. Female (4-week) BALB/c-nu mice were applied to evaluate the inhibitory effect of R59022 on tumor malignancy in immune-deficient mice. The ESCC cell line and the dose of R59022 protocols of BALB/c-nu xenograft mouse model were consistent with those of humanized xenograft mouse model.

MC38 (Cat # ENH2040, RRID: CVCL_B288, Kerafast) or MFC (Cat # TCM23, National Collection of Authenticated Cell Cultures) cells (1 × 10^6^/mouse) were subcutaneously inoculated into the flanks of C57BL/6J (RRID: IMSR_JAX: 000664, female, 4-week) or 615 (RRID: MGI: 2669495, male, 4-week) mice. When tumors reached approximately 100 mm^3^, mice were orally treated with R59022 (25 mg/kg/day) and/or mouse anti-CTLA-4 antibody (50 μg/mouse for up to four times, i.p.; Bio X Cell, Cat # BE0164) biweekly alone, or their combination for 2 weeks. The numbers of CD8^+^ T cells, and levels of PD-L1, CD31, LYVE-1, and NF-κB p65 in local tumor tissues were evaluated using CD8 (Cat # EM2068, FineTest), PD-L1 (Cat # EM0399, FineTest), CD31 (Cat # EM0155, FineTest), LYVE-1 (Cat # EM0593, FineTest), and NF-κB p65 (Cat # EM1231, FineTest) quantitative ELISA assays according to manufacturer’s instructions. Parallelly, MC38 or MFC tumor-bearing mice received R59022 (12.5 mg/kg/day, orally) alone or in combination with anti-CTLA-4 antibody (following the protocol above) for 2 weeks to assess tumor volume changes.

For the IHC assay, slices of MC38 and MFC tumor tissues were deparaffinized and rehydrated. Antigen retrieval was performed by boiling the slices for 15 min in Tris-EDTA buffer. Subsequently, the slices were incubated overnight with antibodies against c-Myc (1:1000), Ki67 (1:500), HMGB1 (1:200), MMP1 (1:100), MMP2 (1:100), MMP7 (1:50), VEGFA (1:100), VEGFC (1:50), CTLA-4 (1:1000) and Foxp3 (1:500) at 4 ℃. The 3, 3′-diaminobenzidene (DAB) was used for staining.

The synergistic interaction between low dose of R59022 and anti-CTLA-4 antibody was assessed using Jin’s formula (Figure S22A, B, C, and D). The Q was derived using the equation Q = Inhibitory effect A + B/(Inhibitory effect A + Inhibitory effect B – Inhibitory effect A × Inhibitory effect B), where Q values > 1.15， 0.85 – 1.15，and < 0.85 were considered synergism, additive effect, and antagonism, respectively.

**Multiplex staining**

Slices of KYSE410 tumors (approximately 5 μm) from formalin-fixed and paraffin-embedded tissue samples were deparaffinized and rehydrated using a graded series of ethanol, followed by antigen retrieval. Multiplex staining was performed with primary antibodies against CD45 (1:100), CD3 (1:100), CD8 (1:100) and GZMB (1:100). After washing with PBS, the slices were incubated with a secondary antibody. Next, DAPI staining solution was applied for nuclear counterstaining, and an antifade mounting medium was used to mount the slices.

**scRNA-Seq analysis**

KYSE410 tumors from control and R59022-treated groups (n = 2/group) were lysed by collagenase and neutral proteinase. The sorted cells were suspended by PBS buffer (containing 0.04% BSA) and were loaded onto a 10 × Chromium chip. Subsequently, cDNA amplification and library construction were conducted, and Illumina NovaSeq 6000 system (RRID: SCR_016387) was employed to sequence the libraries.

Raw scRNA-seq data were aligned to the combined mouse (GRCm39) and human (GRCh38) genomes by Cell Ranger (version 8.0.1, RRID: SCR_017344). The count matrices of results were then processed using the Seurat package (version 5.0.1, RRID: SCR_016341) in R. Cells with low quality were filtered out according to the following criteria: fewer than 400 detected genes, more than 7,500 detected genes, fewer than 1,000 unique molecular identifiers (UMIs), or more than 50,000 UMIs. The remaining count data were log-normalized and scaled to 10,000. The top 3,000 most variable genes were used to perform principal component analysis (PCA). Harmony (version 1.1.0, RRID: SCR_022206) was employed to correct batch effects of the top 20 principal components across different samples. The top 20 dimensions from PCA were used to generate a Uniform Manifold Approximation and Projection (UMAP) plot for data visualization and clusters were determined using the k-nearest neighbor method with a resolution parameter set to 0.3. Human T cell clusters were identified based on the expression of GRCh38-CD3D and further re-clustered into subsets. The scRNA-seq data had been uploaded to National Genomics Data Center Biological Project Library (ID: PRJCA040528).

**Differential expression and enrichment analyses**

GO enrichment analysis for the biological process category was performed on the differentially expressed genes between R59022-treated and control samples using the enrichGO function from the clusterProfiler (version 4.4.4, RRID: SCR_016884) R package. To control the false discovery rate (FDR), *p* values were adjusted using the Benjamini-Hochberg (BH) method. The primary objective of scRNA-seq analysis is to assess the regulatory effect of R59022 on the pathways in T cells.

**IHC staining of gastrointestinal tumors**

All clinical IHC relevant experimental procedures were according to previous study [4]. The dilution of primary antibodies was as follows: DGKα (1: 100), NF-κB p65 (1: 100), or PD-L1 (1: 250). The quantitative analysis of proteins was calculated according to the following formula: staining score = staining intensity (defined as 0 = negative, 1 = weak, 2 = moderate, 3 = strong) × percentage of positive cells area (defined as 0 − 100% = 0 − 100). The primary objective of Figure S1 is to evaluate the correlation between DGKα and PD-L1 across eight gastrointestinal cancers. We performed Z-score normalization within each cancer type and then pooled the normalized data from eight cancer types to analyze the overall correlation between DGKα and PD-L1.

**Evaluation of PA in plasma or tumor tissues and DGKα in tumor tissues from patients with gastrointestinal tumors**

All tumor samples preparation procedures were approved by the Institutional Review Board of Peking University Cancer Hospital & Institute (2024KT176). Informed consent was obtained from all the participants in the study. The antitumor efficacy of ICIs was measured according to response evaluation criteria in solid tumors (RECIST) version 1.1. The responsive group is composed of complete response (CR) and partial response (PR). The non-responsive group includes stable disease (SD) and progressive disease (PD). Plasma or paired tumor tissues from patients with ESCC, gastric cancer and colon cancer were applied to evaluate the concentration of PA in plasma or tumor tissues using a PA assay (Cat # ab273335, Abcam), according to manufacturer’s instructions.

Briefly, the tissues were homogenized with PA assay buffer, lysed, and the plasma was incubated with an organic solvent combination of chloroform/methanol/12N HCl and vortexed. The mixture was centrifuged at 3000 *g* for 10 min at room temperature. The lower organic layer containing solubilized lipids was incubated with a PA converter, reaction mix for 30 min at 37 ℃. PA levels were measured at excitation/emission wavelengths of 535/587 nm.

To assess DGKα levels in tumor tissues, the tissues were homogenized with RIPA buffer, and the protein lysate was collected. DGKα quantitative ELISA kits were applied to evaluate the level of DGKα (Cat # USEC435Hu, USCN) in tumor tissues. Protein lysates from the indicated tumor tissues were incubated in ELISA plates, followed by the sequential addition of primary or secondary antibody solution, TMB solution, and stop solution. The optical value (OD) value was measured at 450 nm. Figure S15 aims to evaluate the correlation between plasma PA and tissue PA and DGKα in responsive groups from ESCC and GC patients (Cohort 1). Data was processed using the same statistical corrections described in Figure S1. In Figure S16, we generated an ROC curve using plasma samples from Cohort 2. We selected a threshold with a sensitivity of at least 90% to effectively minimize false negative.

**nanoDSF assay**

The nanoDSF (Differential Scanning Fluorimetry, Prometheus NT.40, RRID:SCR_025758, Nano Temper) experiment was used to determine the binding of PA to the NF-κB p65 protein (Cat # RP02998, Abclonal). The NF-κB protein was combined with binding buffer. Thereafter, 200 µM PA (Cat # 840857P, Merck) was added to the protein solution to assess the interaction. Subsequently, the mixtures were transferred to capillaries, heated a nanoDSF system, and measured the melting temperature (Tm) values of the NF-κB protein with and without PA.

**BLI assay**

The binding between the biotinylated peptides of NF-κB p65 (synthesized by Suzhou Synbio technologies Company) and PA were detected by BLI assay on an Octet RED96e system (Sartorius). Briefly, biotinylated peptides were synthesized using Fmoc-based solid-phase peptide synthesis. Streptavidin biosensors were preprocessed in PBS buffer for 10 min, followed by the immobilization of indicated biotinylated peptides (10 μg/mL) onto the biosensor. After establishing a stable baseline, the peptide-coated sensors were incubated with PA at different concentrations and then dissociated in PBS buffer. The response data were analyzed, and the PA dissociation constant (*K*_D_) values were calculated by fitting the kinetic curves using Graphpad prism 8.0 (RRID: SCR_002798).

**Molecular docking**

The 3D structure of NF-κB p65 was obtained from RCSB database (PDB ID: 1NFI) and the structure of PA was from PubChem database (Compound CID: 9547158) and converted into a 3D structure in ChemDraw 3D (RRID: SCR_016768). The structure of NF-κB p65 was processed using AutoDock Tools software (RRID: SCR_012746) to perform hydrogenation, delete water, and charge distribution. The structure of PA was energy-minimized using the MMFF94 force field. Then, docking was performed in AutoDock Vina software (RRID: SCR_011958).

**CETSA assay**

KYSE410 and KYSE510 cells were treated with PA (50 μM) and lysed by CETSA cell lysis buffer (Cat # CETSA-BUF2, PerkinElmer) at 4 ℃ for 30 min. The lysate was centrifuged, and the supernatant was incubated at a series of temperatures from 37 to 62 ℃, with an incubation time of 3 min at each temperature point. The expression of protein at each temperature was detected by immunoblotting.

**Luciferase assay**

HEK293T cells (Cat # CRL-3216, RRID: CVCL_0063, ATCC) were co-transfected with pcDNA3.1-*RELA*-Flag plasmid and pGL3-Basic *CD274* promoter region containing GCAAATTCCG sequence or a mutant of this sequence (synthesized by Shanghai Shenggong Biotechnology Company). The activity of NF-κB p65-mediated *CD274* transcriptional activation was evaluated using dual-luciferase reporter assay (Cat # E1910, Promega).

**Pull down assay of biotin-dsDNA**

Streptavidin agarose 6FF beads were preprocessed and allowed to bind to 5 µg biotin-dsDNA (*CD274* promoter region 1; synthesized by Suzhou Synbio technologies Company) for 30 min at room temperature. HEK293T cells were lysed using the cell lysis buffer containing protease and phosphatase inhibitors. After centrifugation, the supernatant was mixed with dsDNA beads overnight at 4 ℃. The beads were then washed, and the loading buffer was added to perform immunoblotting to detect NF-κB p65.

**Statistical analysis**

The Graphpad prism 8.0 and JASP 0.95.4 (RRID: SCR_015823) were used for creating figures and conducting statistical analysis. The unpaired Student’s t-test was applied for analyzing the significant difference between two groups in preclinical experiments and clinical sample assays, and the effect sizes were calculated using absolute of Cohen’s d. The R59022 dose-dependent assays and drug combination studies were statistically analyzed using the one-way analysis of variance (one-way ANOVA) test, followed by Tukey’s post-hoc test. The Pearson correlation coefficient was used for analyzing the correlation between two groups in clinical sample assays and the BH method performed in R 4.4.3 was employed to adjust *p* values in Figure S13 and S17.

**References**

1. Chen, Jie, Di Zhao, Lingyuan Zhang, Jing Zhang, Yuanfan Xiao, Qingnan Wu, Yan Wang. 2024. “Tumor-associated macrophage (TAM)-secreted CCL22 confers cisplatin resistance of esophageal squamous cell carcinoma (ESCC) cells via regulating the activity of diacylglycerol kinase α (DGKα)/NOX4 axis.” *Drug Resistance Updates* 73:101055. <https://doi.org/10.1016/j.drup.2024.101055>

2. Cha, Jong-Ho, Wen-Hao Yang, Weiya Xia, Yongkun Wei, Li-Chuan Chan, Seung-Oe Lim, Chia-Wei Li. *et al*. 2018. “Metformin promotes antitumor immunity via endoplasmic-reticulum-associated degradation of PD-L1.” *Molecular Cell* 71:606−620.e7. <https://doi.org/10.1016/j.molcel.2018.07.030>

3. Lau, Poyee, Guanxiong Zhang, Shuang Zhao, Long Liang, Hailun Zhang, Guowei Zhou, Mien-Chie Hung. *et al*. 2022. “Sphingosine kinase 1 promotes tumor immune evasion by regulating the MTA3-PD-L1 axis.” *Cellular & Molecular Immunology* 19:1153−1167. <https://doi.org/10.1038/s41423-022-00911-z>

4. Chen, Jie, Yan Wang, Weimin Zhang, Di Zhao, Lingyuan Zhang, Jing Zhang, Jiawen Fan. *et al*. 2021. “NOX5 mediates the crosstalk between tumor cells and cancer-associated fibroblasts via regulating cytokine network.” *Clinical Translational Medicine* 11:e472. <https://doi.org/10.1002/ctm2.472>

**Supplementary Figures and figure legends**

**Figure S1 The clinical correlation between DGKα and PD-L1 in gastrointestinal cancers**

(A) The experimental process of detecting correlation between DGKα and PD-L1 in gastrointestinal cancer tissue by IHC assay. (B) IHC staining results of DGKα and PD-L1 expression in 8 types of gastrointestinal cancers, including ESCC (6 independent cases), adenocarcinoma of the gastroesophageal junction (AEG, 6 independent cases), gastric cancer (GC, 7 independent cases), colon cancer (CC, 5 independent cases), rectal cancer (RC, 5 independent cases), hepatocellular carcinoma (HCC, 6 independent cases), pancreatic cancer (PCA, 7 independent cases), and gallbladder cancer (GBC, 6 independent cases). (C) The Pearson correlation coefficient between DGKα IHC score and PD-L1 IHC score in 8 gastrointestinal cancers was indicated. *p* value is shown.

**Figure S2 DGKα Inhibition blocks PD-L1 expression and enhances the tumor-inhibitory effect of T cells**

(A−B) Results from quantitative ELISA (A) and immunoblotting (B) assays show that R59022 (25 µM, 50 µM) inhibits the expression of PD-L1 in KYSE410, KYSE510, and HCT116 cells. (C) The knockdown efficacy of DGKα is evaluated using immunoblotting assay. (D−E) Results from quantitative ELISA (D) and immunoblotting (E) assays show that knocking down *DGKA* by shRNAs blocks the expression of PD-L1 and adding PA (50 µM) restores the expression of PD-L1 in KYSE410, KYSE510, and HCT116 cells. (F) R59022 (50 µM) enhances the cytotoxicity of T cells in indicated gastrointestinal tumor cells. Data are represented as mean ± SD of n = 3 (A, D) and 5 (F) biologically independent samples. Statistical significance for (A, F) or (D) was respectively determined by one-way ANOVA, followed by Tukey’s post-hoc test or unpaired Student’s t-test. *p* values and size effect (Cohen’s d, marked in red) are shown.

**Figure S3 Diagram showing the approach and protocol to validate PA-induced NF-κB p65 transcriptional activity**

ChIP and ELISA assays are used to identify the interaction between *CD274* promoter and NF-κB p65 and detect the NF-κB p65-mediated PD-L1 expression (left). Molecular docking, CETSA, BLI and nanoDSF assays are employed to identify the directly binding between PA and NF-κB p65 (middle). Biotin-dsDNA pull down assay and luciferase assay are used to observe that PA-bound to NF-κB p65 to enhance its-mediated transcriptional activation of PD-L1 (right).

**Figure S4 NF-κB is critical for PA-mediated PD-L1 expression**

(A) The knockdown efficacy of NF-κB p65 is evaluated using immunoblotting assay. (B) KYSE410 and KYSE510 cells harboring *RELA* siRNAs are treated with PA (50 µM) and the expression of PD-L1 is assessed using immunoblotting assay.

**Figure S5 PA interacts with the specific sites of NF-κB p65**

(A) CETSA results show PA (50 µM) cannot interact with NF-κB p65 in KYSE410 cells harboring H181A, P182A, F184A, P189A, Q220A, K221A, or R274A mutant from 37 ℃ to 62 ℃. (B) BLI results show that the interaction statuses between PA and indicated peptides. *K*_D_ values are indicated.

**Figure S6 The growth inhibitory effect of R59022 on the KYSE410 tumor in BALB/c-nu mice**

(A) Changes of tumor volume within 3 weeks in R59022 (25 mg/kg)-treated BALB/c-nu mice harboring KYSE410 tumors (n = 5/group). (B) The tumor volume on the last day is indicated. Data are represented as mean ± SD of n = 5 (A, B) biologically independent samples. Statistical significance for (B) was determined by unpaired Student’s t-test. *p* value and size effect (Cohen’s d, marked in red) are shown.

**Figure S7 Differential expression of tumor-promoting genes between control and R59022-treated KYSE410 tumor**

(A) Results from scRNA-seq show that R59022 suppresses the expression of representative proliferation-related genes (Purple) and NF-κB-controlled genes (Black). (B) Gene set enrichment analysis (GSEA) results indicate that the pathway of cell proliferation is significantly downregulated in the R59022-treated tumor (Normalized enrichment score (NES) = -1.5, *p*_adj_ = 0.01).

**Figure S8 Seven NK/T cell subsets clustering and markers expression in NK/T cell subsets**

(A) Results from scRNA-seq of R59022-treated and control tumors (n = 2/group) show that seven NK/T cell were clustered. (B) The seven NK/T cells were identified based on known markers: *TIGIT* for Tex, *neural cell adhesion molecule 1* (*NCAM1*) for NK cells, *FOXP3* for Treg, *GZMK* and *GZMH* for Teff, *IL7R* for Tem, *T-cell factor 7* (*TCF7*) for naïve T cells, and *MKI67* for proliferating T cells.

**Figure S9 R59022 inhibits the expression of Tex biomarker-*TIGIT***

Differential expression analysis results show that R59022 inhibits the expression of Tex biomarker-*TIGIT*. *p* value is calculated with Wilcoxon test (two-sided).

**Figure S10 Downregulation of glucose metabolism-related pathways and genes in R59022-treated Treg**

(A) GO enrichment results show that R59022 inhibits the glucose metabolism-related pathways in Treg. (B) Differential expression analysis results show that the glycolysis-related genes are downregulated in R59022-treated Treg.

**Figure S11 Upregulation of IFN-related pathways and genes in R59022-treated myeloid cells**

(A) GO enrichment results show that R59022 promotes the IFN-related pathways in myeloid cells. (B) Differential expression analysis results show that the IFN-related genes *ISG15*, *interferon-inducible protein 6* (*IFI6*), and *2'-5' oligoadenylate synthetase 3* (*OAS3*) are significantly upregulated in R59022-treated myeloid cells. *p* values are calculated with Wilcoxon test (two-sided).

**Figure S12 PKCζ is not involved in the DGKα/PA axis-mediated PD-L1 expression**

(A) KYSE410 (Left panel) and KYSE510 (Right panel) cells are treated with or without PA (50 μM), and the lysates are immunoprecipitated with NF-κB p65 antibody. Then samples are subjected to immunoblotting to evaluate the expression of PKCζ and NF-κB p65. (B−C) KYSE410 (Left panel) and KYSE510 (Right panel)-incubated with PA (50 µM) are in the presence or absence of PKCζ inhibitor-ζ-Stat (1 μM). The PD-L1 expression is assessed using immunoblotting assay (B), and the interaction between NF-κB p65 and *CD274* promoter region is evaluated using ChIP assay (C). Data are represented as mean ± SD of n = 3 biologically independent samples. ns no significant difference.

**Figure S13 The expression of PA in plasma and paired tumor tissues and the correlation between plasma PA and tissue DGKα/PA axis in ICIs-treated gastrointestinal cancers**

(A) Diagram shows the experimental process of detecting DGKα/PA/NF-κB axis in ICIs responsive and non-responsive gastrointestinal tumor plasma and tissues. (B−C) Results from quantitative ELISA assay show PA levels in ICIs responsive gastrointestinal cancer plasma (ESCC 57 independent cases; colon cancer, CC 39 independent cases; gastric cancer, GC 58 independent cases), non-responsive gastrointestinal cancer plasma (ESCC 62 independent cases; CC 52 independent cases; GC 130 independent cases) (B), responsive gastrointestinal cancer paired tissues (ESCC 8 independent cases, CC 2 independent cases, GC 6 independent cases) and non-responsive paired tissues (ESCC 13 independent cases, CC 10 independent cases, GC 42 independent cases) (C). (D) Correlation between plasma PA and paired tissue PA (upper panel), plasma PA and paired tissue DGKα (middle panel), tissue PA and tissue DGKα (lower panel) in non-responsive gastrointestinal cancer (ESCC 13 independent cases, CC 10 independent cases, GC 42 independent cases). Statistical significance for (B, C) or (D) was respectively determined by unpaired Student’s t-test or Pearson correlation analysis, followed by BH correction. *p* and adjust *p* values, and size effect (Cohen’s d, marked in red) are indicated.

**Figure S14 The expression of** **DGKα in tumor tissues in ICIs-treated responsive and non-responsive gastrointestinal cancers**

Results from quantitative ELISA assay show that DGKα levels in ICIs responsive gastrointestinal cancer tissues (ESCC 8 independent cases, CC 2 independent cases, GC 6 independent cases) and non-responsive tissues (ESCC 13 independent cases, CC 10 independent cases, GC 42 independent cases). Statistical significance was determined by unpaired Student’s t-test. *p* values and size effect (Cohen’s d, marked in red) are indicated.

**Figure S15 Correlation between plasma PA, tissue PA and tissue DGKα in ICIs-treated responsive ESCC and GC patients**

(A−C) Correlation between plasma PA and paired tissue PA (A), plasma PA and paired tissue DGKα (B), tissue PA and tissue DGKα (C) in responsive ESCC 8 independent cases and GC 6 independent cases. The correlations are analyzed by Pearson correlation coefficient. *p* values are indicated.

**Figure S16 ROC curve shows the effect of plasma PA on evaluating immunotherapy response**

(A−C) ROC curves show the performance of plasma PA (Cohort 2) on evaluating immunotherapy response in ESCC patients (Area under the curve (AUC) = 0.945, A), colon cancer patients (AUC = 0.9672, B), and gastric cancer patients (AUC = 0.9751, C).

**Figure S17 The expression of** **DGKα and** **NF-κB in ICIs-treated responsive and non-responsive gastrointestinal tumor tissues and the correlation between DGKα and NF-κB in non-responsive gastrointestinal tumor tissues**

(A) Results from IHC assay show the expression of DGKα and NF-κB in ICIs-treated responsive and non-responsive ESCC, CC and GC tissues. (B) The IHC score of DGKα and NF-κB in responsive (8 independent cases) and non-responsive (13 independent cases) ESCC tissues, responsive (2 independent cases) and non-responsive (10 independent cases) CC tissues and responsive (6 independent cases) and non-responsive (42 independent cases) GC tissues. (C) Correlations between DGKα and NF-κB in ICIs-treated non-responsive ESCC (13 independent cases), CC (10 independent cases) and GC (42 independent cases) tissues. Statistical significance for (B) or (C) was respectively determined by unpaired Student’s t-test or Pearson correlation analysis, followed by BH correction. *p* and adjust *p* values, and size effect (Cohen’s d, marked in red) are indicated.

**Figure S18 CD8 and PD-L1 levels in MC38 or MFC tumor tissues from R59022-treated MC38 and MFC subcutaneous tumor-bearing mice model**

Results from quantitative ELISA assay show that R59022 induces the infiltration of CD8^+^ T cells and inhibits the expression of PD-L1 in MC38 (A) and MFC (B) tumor tissues in C57BL/6J and 615 mice. Data are represented as mean ± SD of n = 3 (A, B) biologically independent samples. Statistical significance for (A, B) was determined by unpaired Student’s t-test. *p* values and size effect (Cohen’s d, marked in red) are shown.

**Figure S19 Combined administration of R59022 and anti-CTLA-4 antibody in MC38 and MFC subcutaneous tumor-bearing mice model**

(A) Diagram shows that MC38 and MFC cells were used to establish subcutaneous tumor-bearing C57BL/6J and 615 mouse models. R59022 (25 mg/kg/day, orally) and anti-CTLA-4 antibody (50 μg/mouse for up to four times, i.p.) were co-administrated and the tumor tissues were collected to detect the tumor volume and markers. (B) The change of MC38 and MFC tumor volume in R59022-treated and anti-CTLA-4 antibody-treated groups or their combination. The tumor volume of last day is indicated. Data are represented as mean ± SD of n = 5 (B) biologically independent samples. (C) Results from quantitative ELISA assay show NF-κB, CD31 and LYVE-1 expressions in MC38 and MFC tumor tissues. (D) Results from IHC assay show the levels of tumor markers, including proliferation markers c-Myc and Ki67, metastasis markers HMGB1, MMP1, MMP2, MMP7, VEGFA, and VEGFC in MC38 tumors. Data are represented as mean ± SD of n = 3 (C, D) biologically independent samples. Statistical significance was determined by one-way ANOVA, followed by Tukey’s post-hoc test. *p* values and size effect (Cohen’s d, marked in red) are indicated.

**Figure S20 The change of tumor volume in MC38 and MFC subcutaneous tumor-bearing mice model**

(A−B) The change of MC38 (A) and MFC (B) tumor volume of each mouse in R59022 (25 mg/kg)-treated and anti-CTLA-4 antibody (50 µg/mouse)-treated groups or their combination.

**Figure S21 Tumor markers expression in MFC subcutaneous tumor-bearing mice model tumor tissues**

Results from IHC assay show the levels of tumor-promoting markers, including proliferation markers c-Myc and Ki67, metastasis markers HMGB1, MMP1, MMP2, MMP7, VEGFA, and VEGFC in MFC tumors. Data are represented as mean ± SD of n = 3 biologically independent samples. Statistical significance was determined by one-way ANOVA, followed by Tukey’s post-hoc test. *p* values and size effect (Cohen’s d, marked in red) are indicated.

**Figure S22 Combined administration of low dose R59022 and anti-CTLA-4 antibody in MC38 and MFC subcutaneous tumor-bearing mice model**

(A−B) The change of MC38 (A) and MFC (B) tumor volume in R59022 (12.5 mg/kg)-treated and anti-CTLA-4 antibody (50 µg/mouse)-treated groups or their combination. The tumor volume of last day is indicated. (C−D) The growth of each mouse harboring MC38 (C) and MFC (D) tumors in R59022 (12.5 mg/kg)-treated and anti-CTLA-4 antibody (50 µg/mouse)-treated groups or their combination. Data are represented as mean ± SD of n = 5 biologically independent samples (A, B). (E) The IHC results show that Treg (marked by Foxp3), Ki67 and CTLA-4 expressions in indicated groups in MC38 and MFC tumors. Data are represented as mean ± SD of n = 3 biologically independent samples (E). Statistical significance was determined by one-way ANOVA, followed by Tukey’s post-hoc test. *p* values and size effect (Cohen’s d, marked in red) are indicated.

**Figure S23 The combination of R59022 and anti-CTLA-4 antibody does not induce obvious systemic toxicity in xenograft mouse model**

(A−B) H&E staining of kidney, spleen, liver, and heart tissues from mice harboring MC38 (A) or MFC (B) xenografts treated with indicated agents.
